# ELAC2/RNaseZ-linked cardiac hypertrophy in *Drosophila melanogaster*

**DOI:** 10.1242/dmm.048931

**Published:** 2021-08-31

**Authors:** Ekaterina Migunova, Joanna Theophilopoulos, Marisa Mercadante, Jing Men, Chao Zhou, Edward B. Dubrovsky

**Affiliations:** 1 Department of Biological Sciences, Fordham University, Bronx, NY 10458, USA; 2 Department of Biomedical Engineering, Washington University in St Louis, St Louis, MO 63105, USA; 3 Department of Electrical and Computer Engineering, Lehigh University, Bethlehem, PA 18015, USA; 4 Center for Cancer, Genetic diseases, and Gene Regulation, Department of Biological Sciences, Fordham University, Bronx, NY 10458, USA

**Keywords:** *Drosophila*, Cardiomyopathy, ELAC2, RNase Z, OCM

## Abstract

A severe form of infantile cardiomyopathy (CM) has been linked to mutations in *ELAC2*, a highly conserved human gene. It encodes Zinc phosphodiesterase ELAC protein 2 (ELAC2), which plays an essential role in the production of mature tRNAs. To establish a causal connection between ELAC2 variants and CM, here we used the *Drosophila melanogaster* model organism, which carries the ELAC2 homolog RNaseZ. Even though RNaseZ and ELAC2 have diverged in some of their biological functions, our study demonstrates the use of the fly model to study the mechanism of ELAC2-related pathology. We established transgenic lines harboring RNaseZ with CM-linked mutations in the background of endogenous RNaseZ knockout. Importantly, we found that the phenotype of these flies is consistent with the pathological features in human patients. Specifically, expression of CM-linked variants in flies caused heart hypertrophy and led to reduction in cardiac contractility associated with a rare form of CM. This study provides first experimental evidence for the pathogenicity of CM-causing mutations in the ELAC2 protein, and the foundation to improve our understanding and diagnosis of this rare infantile disease.

This article has an associated First Person interview with the first author of the paper.

## Abstract (precis)

**Summary:** A newly established *Drosophila* model recapitulates key features of human heart pathology linked to mutations in *ELAC2*, thus providing experimental evidence of the pathogenicity of ELAC2 variants.

## INTRODUCTION

Cardiomyopathy (CM) is a group of diseases that weaken the heart muscle and compromise its ability to pump blood. Cardiomyopathies can lead to major health complications, such as arrhythmia, heart failure and sudden cardiac death. Two main types of CM are recognized based on heart morphology and function: hypertrophic cardiomyopathy (HCM) and dilated cardiomyopathy (DCM). The most prevalent form is HCM, with one in 500 people being affected. It is defined by the thickening of the left ventricular heart wall, cardiomyocyte hypertrophy, and reduced heart volume at systole and diastole. HCM is the most common cause of sudden cardiac death among adolescent children (McKenna et al., 1981). DCM, however, is estimated to occur in one out of 2500 people. DCM is described as the dilation of either the left or both ventricles together with systolic dysfunction (Taylor et al., 2006). In rare cases, HCM can progress into dilated stage (D-HCM), which combines both heart wall thickening and heart cavity dilation. D-HCM has a very poor prognosis and often ends with heart failure, whereas DCM can be managed (Hamada et al., 2010). Despite good comprehension of CM pathophysiology, the molecular mechanisms leading to this condition are not completely understood, nor have the effective prevention strategies been developed yet.

Cardiomyopathies affect people of all ages. The number of CM cases in infants is lower than in adults but the mortality rate among newborns is significantly higher, especially if the symptoms develop before the age of 1 (Maron et al., 1995). Unlike the adult form, which is primarily caused by particular health conditions, viral infections and behavior (Guzzo-Merello et al., 2014; Kearney et al., 2001; Messerli et al., 2017), infantile CM is due to inherited mutations; it is clinically and etiologically heterogeneous. At least 80 genes have been linked to infantile cardiomyopathies (Dellefave and McNally, 2010; Roma-Rodriguez and Fernades, 2014); they have been grouped based on the frequency of mutations associated with infantile CM (Hershberger et al., 2013; Keren et al., 2008; McNally et al., 2015). Most often affected are genes that encode proteins involved in cardiomyocyte contraction through their association with sarcomeres, e.g. cardiac myosins and troponins (Hershberger et al., 2013; Kamisago et al., 2000). The second group of genes that are frequently impaired in CM patients encode cytoskeleton proteins in myocytes, e.g. vinculin (Olson et al., 2002) and cypher (LDB3) (Vatta et al., 2003). Finally, there is a large third group of genes, whose estimated contribution to the total number of CM cases is relatively small. Genes of this group encode a variety of different proteins, and include ion channel, cell junction, spliceosome and nuclear envelope proteins to name a few (Hershberger et al., 2013).

A recent discovery added another gene to the list of those associated with infantile CM. Mutations in the gene encoding the tRNA processing endonuclease (*ELAC2*, hereafter referred to as RNaseZ) are linked to the early onset of CM symptoms (Haack et al., 2013; Shinwari et al., 2017). Patients diagnosed with cardiomyopathy due to mutations in *ELAC2* have a median life expectancy of 4 months (Shinwari et al., 2017). *ELAC2*-related heart impairment has allelic and phenotypic heterogeneity. Together, 25 *ELAC2* alleles have been associated with cardiac pathology: two frameshift, five splice and 18 missense mutations (Saoura et al., 2019). In addition to CM, patients carrying *ELAC2* mutations suffer from a variety of abnormalities including mitochondrial respiratory chain deficiency, retardation of the intrauterine growth, delay in the psychomotor development, encephalopathy and muscular hypotonia (Akawi et al., 2016; Haack et al., 2013; Kim et al., 2017; Shinwari et al., 2017). Most patients with *ELAC2* mutations were diagnosed with HCM; although, there were few cases described as DCM or D-HCM (Haack et al., 2013; Shinwari et al., 2017; Saoura et al., 2019). Although advanced genetic studies that involve exome sequencing and pedigree analysis found a strong association between ELAC2 variants and CM (Haack et al., 2013), experimental evidence providing proof of the disease-causing effect of *ELAC2* mutations is still missing. To experimentally validate the predicted damaging effect of ELAC2 variants a well-established model organism can be used.

**Fig. 1.**
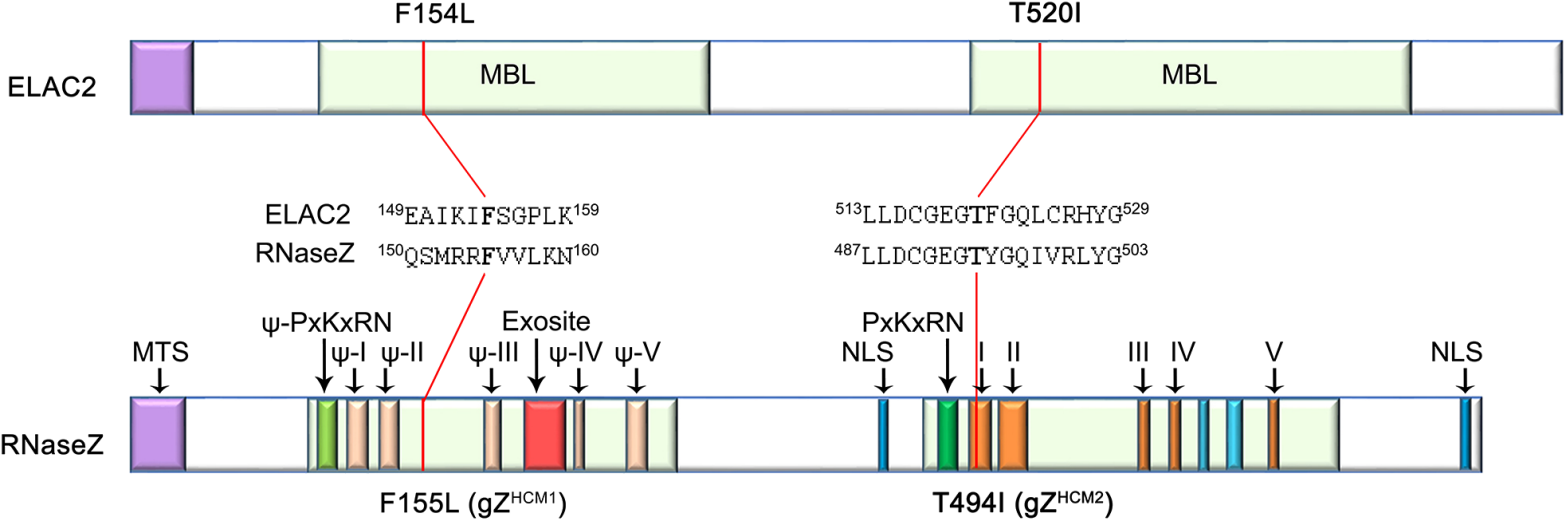
**Comparison of human ELAC2 and *Drosophila* RNaseZ proteins.** Two colored bars represent protein structures of ELAC2 and RNaseZ with conserved MBL domains and functional motifs being designated (drawn not to scale). Localization and conservation of amino acid residues known to be affected by CM-linked mutations – F154L, T520I – as well as the positions of mutations – F155L, T494I – introduced into RNaseZ amino acid sequence are shown. MTS, mitochondrial targeting sequence; NLS, nuclear localization signal.

The ELAC2 protein has homologs in all eukaryotes, and its function has been studied extensively by using the *Drosophila melanogaster* homolog RNaseZ (Dubrovsky et al., 2004). ELAC2/RNaseZ endoribonuclease is essential for tRNA maturation. Initially, a tRNA is produced with extensions at both ends (Phizicky and Hopper, 2010). For the tRNA molecule to become functional endonucleolytic cuts have to be made by RNaseP and RNaseZ at the 5′ and 3′ end, respectively (Hartmann et al., 2009). RNaseZ belongs to the metallo-β-lactamase (MBL) family of proteins, all of which feature a common structure, the MBL domain (Kostelecky et al., 2006). This domain is composed of five motifs (I to V). Motifs II, III, IV and V comprise seven highly conserved histidine and/or aspartate residues involved in zinc ion binding and coordination within the catalytic center (Aravind, 1999; Vogel et al., 2002; Späth et al., 2005). Motif I, while being part of the active site of the enzyme, is also involved in substrate recognition and binding. RNaseZ has two additional functional motifs – a PxKxRN loop and an exosite both contributing to the tRNA substrate recognition (Kostelecky et al., 2006; Zareen et al., 2006). The tRNA 3′-end processing endonuclease exists in two forms – the short form (RNaseZ^S^), found in all three domains of life, and the long form (RNaseZ^L^) found only in eukaryotes (Vogel et al., 2005). *RNaseZ^L^* is thought to have evolved from the *RNaseZ^S^* gene duplication (Tavtigian et al., 2001; Vogel et al., 2005). Some eukaryotes (*S. cerevisiae*, *C. elegans*, *D. melanogaster*) have only a long version of RNaseZ, while others, e.g. *H. sapiens*, have both forms of the enzyme. In humans, RNaseZ^S^ is encoded by *ELAC1* and RNaseZ^L^ by *ELAC2* (Takahashi et al., 2008). As a consequence of tandem duplication and fusion, RNaseZ^L^ is built of two homologous halves, each carrying the MBL domain. However only the C-terminal domain is catalytically active. Motifs I-V of the N-terminal domain are termed pseudo motifs and do not participate in catalysis (Fig. 1) (Zhao et al., 2010). Interestingly, the exosite is present only in the N-terminal half; the C-terminal half of RNaseZ^L^ appears to have lost this motif (Ma et al., 2017). Even though the biochemical activity and the crystal structure of functional domains of RNaseZ^L^ protein have been identified (Ma et al., 2017), the understanding of how mutations in the gene encoding this protein lead to cardiac hypertrophy is still missing.

Given the homology of ELAC2 and RNaseZ, we decided to turn to a *Drosophila* model to study the effect of mutant RNaseZ forms on fly heart and body physiology. The fly heart has a tubular structure and is often referred to as the dorsal vessel owing to its spatial position under the dorsal body wall. It is formed during embryogenesis by 104 contractile cardiomyocytes that are arranged in two opposing rows that form a luminal space in between them. The contractile elements of cardiomyocytes are myofibrils, which are discrete bundles of actomyosin filaments, uniformly distributed around the vessel. The dorsal vessel is divided into the heart, a contractile chamber and the aorta, i.e. an outflow tract (Curtis et al., 1999). During embryonic and larval stages, the heart is positioned in the posterior abdominal segments A5-A8 and the aorta extends from the heart towards the anterior end into thoracic segment T3. During metamorphosis, parts of the larval heart undergo programmed cell death, reducing the number of cardiomyocytes to 84 (Sellin et al., 2006). At the same time, the anterior part of the aorta enlarges to form the contractile chamber of an adult heart in abdominal segments A1-A4 (Sellin et al., 2006). Unlike in vertebrates, the heart function in *Drosophila* can be severely compromised without causing immediate death (Zhu et al., 2017a,b). However, the genetic network controlling cardiac development as well as certain features of heart physiology and function are well conserved from flies to mammals (Ocorr et al., 2007). A variety of resources available for *Drosophila* research allow the investigation of cellular and molecular mechanisms that contribute to cardiomyopathies (Wolf and Rockman, 2011; Neely et al., 2010), which makes *Drosophila* a great model to study human cardiac disorders.

Here, we tested whether two missense mutations that were previously identified in human patients (Haack et al., 2013) cause cardiac pathology in *Drosophila*. We created fly models harboring mutated versions of the *RNaseZ* gene that are linked to CM-causing mutations of *ELAC2* and found that these flies did recapitulate main symptoms, including increased thickness of the heart wall, dilated heart lumen and decreased cardiac contractility. These findings provide experimental evidence of a direct connection between ELAC2 variants and cardiomyopathy.

## RESULTS

### Functional homology of human ELAC2 and *Drosophila* RNaseZ proteins

**Fig. 2.**
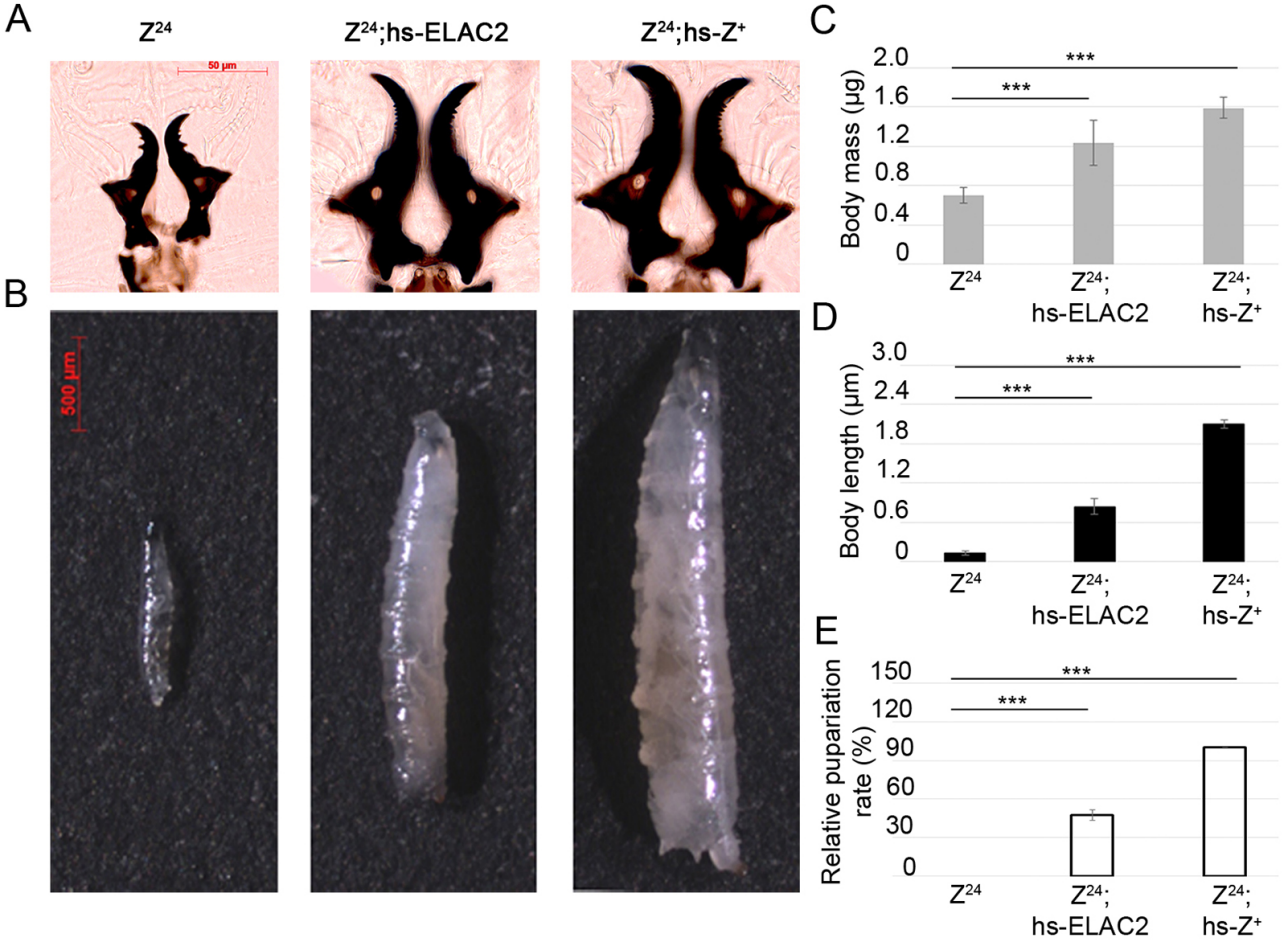
**ELAC2 rescues the early-lethality phenotype of RNaseZ KO homozygotes**. (A) Mouth hook morphology of homozygote KO (Z^24^) and rescued (Z^24^;hs-ELAC2 and Z^24^;hs-Z^+^) larvae shows that both transgenes (hs-ELAC2 and hs-Z^+^) help Z^24^ mutants to survive early lethality and reach 3rd instar. (B) Appearance of the Z^24^ knockout (left panel) at its terminal larval stage compared with rescued larvae Z^24^;hs-ELAC2 (middle panel) and Z^24^;hs-Z^+^ (right panel). (C) Body mass of homozygote KO (Z^24^) and rescued (Z^24^;hs-ELAC2 and Z^24^;hs-Z^+^) animals at their terminal larval stages (*n*=20). (D) Body length of homozygote KO (Z^24^) and rescued (Z^24^;hs-ELAC2 and Z^24^;hs-Z^+^) animals at their terminal larval stages (*n*=20). (E) Pupariation rate of homozygote KO (Z^24^) animals rescued with either hs-ELAC2 or hs-Z^+^ (*n*=240). Z^24^ larvae do not pupariate as they die at 2nd instar. ***P<0.001 (ANOVA). Error bars indicate the mean±s.e.m.

The homology between the human ELAC2 and *Drosophila* RNaseZ proteins was established based on amino acid sequence similarity (Fig. 1) (Dubrovsky et al., 2004; Zhao et al., 2010). Moreover, both proteins have the same biochemical activity *in vitro* and *in vivo* (Takaku et al., 2003; Xie et al., 2011). Here, we decided to evaluate the degree of biological function conservation of ELAC2 and RNaseZ through the rescue experiment. We have previously generated and characterized the Z^24^ null lethal allele of *RNaseZ* (Xie et al., 2013). Z^24^ homozygotes die during 2nd instar on day 3 after egg deposition (AED). We assessed whether the early larval lethality of Z^24^ animals can be rescued by a heat shock (hs)-driven *ELAC2* transgene (hs-ELAC2). As a positive control, we used the wild-type *RNaseZ* transgene under the hs promoter (hs-Z^+^) (Xie et al., 2013). Experimental and control larvae were subjected to hs treatment every 24 h starting at day 1 AED. We found that Z^24^;hs-ELAC2 transgenic flies, indeed, escaped early lethality of the Z^24^ null mutant. On day 5 AED they reached 3rd instar (Fig. 2A); however, there was a clear difference in body size and weight between rescued individuals of two genotypes. Larvae expressing hs-ELAC2 showed, on average, only 77% of the length and 40% of the mass of age-matched control larvae expressing hs-Z^+^ (Fig. 2B-D). hs-ELAC2 larvae exhibited reduced development rate as they pupariated 4 days later than expected. The number of larvae reaching pupariation (59 out of 240) was reduced by 53% compared to control larvae (Fig. 2E). Animals that pupariated died soon after, the rest of larvae survived up to 25 days but eventually died without any sign of pupariation. These results showed that human *ELAC2* introduced into an *RNaseZ* knockout background significantly improves larval viability. However, the pupariation rate of rescued larvae was only 50% compared with that of control larvae, and adult survival is zero. Thus, although the two proteins are known to be true functional homologs, ELAC2 cannot complement RNaseZ knockout in flies.

### Generation of flies carrying CM-linked mutations

Because ELAC2 failed to rescue Z^24^ KO, we decided to use the *Drosophila* homolog RNaseZ to model CM-related mutations in flies. Alignment of ELAC2 and RNaseZ showed that, out of 13 ELAC2 missense mutations associated with CM, seven affect amino acid residues that are highly conserved and located within the same motifs that constitute homologous functional domains of human and *Drosophila* protein. Two of these mutations, F154L and T520I are well characterized in terms of their clinical manifestation (Haack et al., 2013).

Previously, we have described the gZ^+^-V5 construct containing a fragment of genomic DNA that encompasses the coding sequence of V5-tagged RNaseZ together with its endogenous regulatory sequences (Xie et al., 2013). By using this construct and site-directed mutagenesis, we introduced CM-linked missense mutations F155L (nucleotide 463C→T) and T494I (nucleotide 1539C→T) into the *RNaseZ* ORF to produce gZ^HCM1^ and gZ^HCM2^ alleles (Fig. 1). Both mutations were confirmed by DNA sequencing. Western blot analysis showed that transgenic flies carrying corresponding constructs express similar amounts of wild-type and mutant versions of RNaseZ protein (Fig. S2). To study the impact of CM-linked mutations on flies, we introduced gZ^HCM1^ and gZ^HCM2^ transgenes into the *RNaseZ* null background (Z^24^). Thus, transgenes became the sole source of RNaseZ protein. In all experiments we used flies that carry either one (Z^24^;gZ^HCM^/+) or two (Z^24^;gZ^HCM^/gZ^HCM^) copies of the transgene – hereafter designated as 1xgZ^HCM^ and 2xgZ^HCM^, respectively. Control flies carry one copy of the transgene encoding wild-type protein, 1xgZ^+^ (Z^24^;gZ^+^/+).

**Fig. 3.**
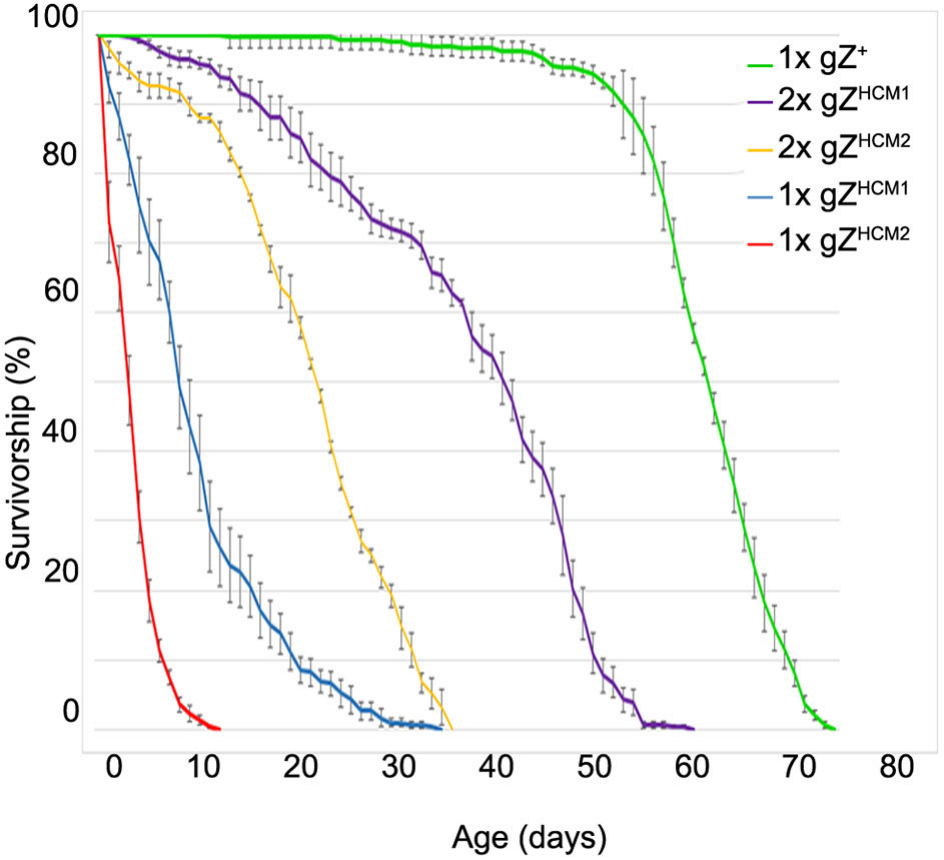
**gZ^HCM^ mutants show a decrease in longevity.** Adult fly longevity was assessed by daily counting live flies over time. Shown are survival rates for Z^24^ KO flies carrying one or two copies of the gZ^HCM^ transgene, i.e. 1xgZ^HCM1^ (Z^24^;gZ^HCM1^/+), 1xgZ^HCM2^ (Z^24^;gZ^HCM2^/+), 2xgZ^HCM1^ (Z^24^;gZ^HCM1^/gZ^HCM1^) and 2xgZ^HCM2^ (Z^24^;gZ^HCM2^/gZ^HCM2^). Control flies carry one copy of the wild-type transgene 1xgZ^+^ (Z^24^;gZ^+^/+). *n*>300 for each genotype. *P*<0.0001 (Mantel–Cox test). Error bars indicate the mean±s.e.m.

*RNaseZ* is an essential gene, which implies that gZ^HCM^ mutations could have an effect on fly development and lifespan. We found that both transgenic fly lines displayed a minor delay regarding their progression through the life cycle, as they pupariated and eclosed 12-24 h later compared to control flies. Interestingly, although the viability of mutant flies at eclosion was not affected, longevity of adults was significantly reduced. The median adult lifespan was ∼41 days for 2xgZ^HCM1^, 22 days for 2xgZ^HCM2^, 9 days for 1xgZ^HCM1^ and 4 days for 1xgZ^HCM2^, compared to 61 days for 1xgZ^+^ control flies (*P*>0.0001; Fig. 3).

**Fig. 4.**
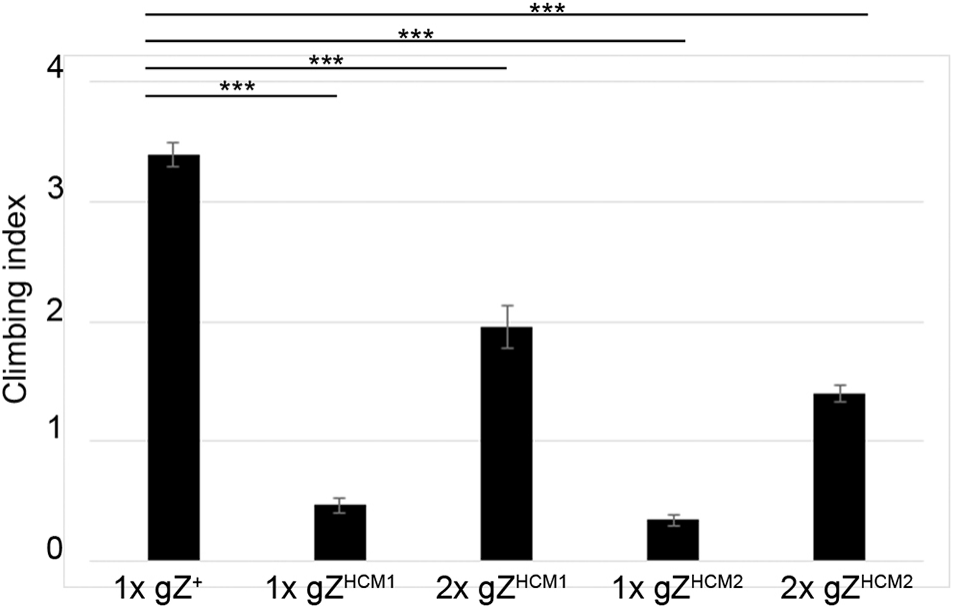
**gZ^HCM^ mutants show a decrease in locomotor response.** Negative geotaxis expressed as the climbing index is shown for control flies 1xgZ^+^ (Z^24^;gZ^+^/+) and mutants – 1xgZ^HCM1^ (Z^24^;gZ^HCM1^/+), 1xgZ^HCM2^ (Z^24^;gZ^HCM2^/+), 2xgZ^HCM1^ (Z^24^;gZ^HCM1^/gZ^HCM1^) and 2xgZ^HCM2^ (Z^24^;gZ^HCM2^/gZ^HCM2^), with *n*>80 for each genotype. ***P<0.001 (one-tailed *t*-test). Error bars indicate the mean±s.e.m.

From day of eclosion, the gZ^HCM^-expressing transgenic flies did not appear as active as age-matched control flies. To evaluate the effect of HCM mutations on fly fitness, we measured the locomotor activity of 2-day-old transgenics by using the negative geotaxis climbing assay (Gargano et al., 2005; Piazza et al., 2009). All flies carrying mutant *RNaseZ* transgenes exhibited a reduction of the climbing index by 47% for 2xgZ^HCM1^, 67% for 2xgZ^HCM2^, 78% for 1xgZ^HCM1^ and 88% for 1xgZ^HCM2^ compared to age-matched control (Fig. 4). In both longevity and fitness assays, flies that carried one copy of the gZ^HCM^ transgene showed a stronger phenotype than flies that carried two copies; therefore, only the former flies were used in subsequent experiments.

### gZ^HCM1^ and gZ^HCM2^ mutations lead to heart hypertrophy in *Drosophila*

**Fig. 5.**
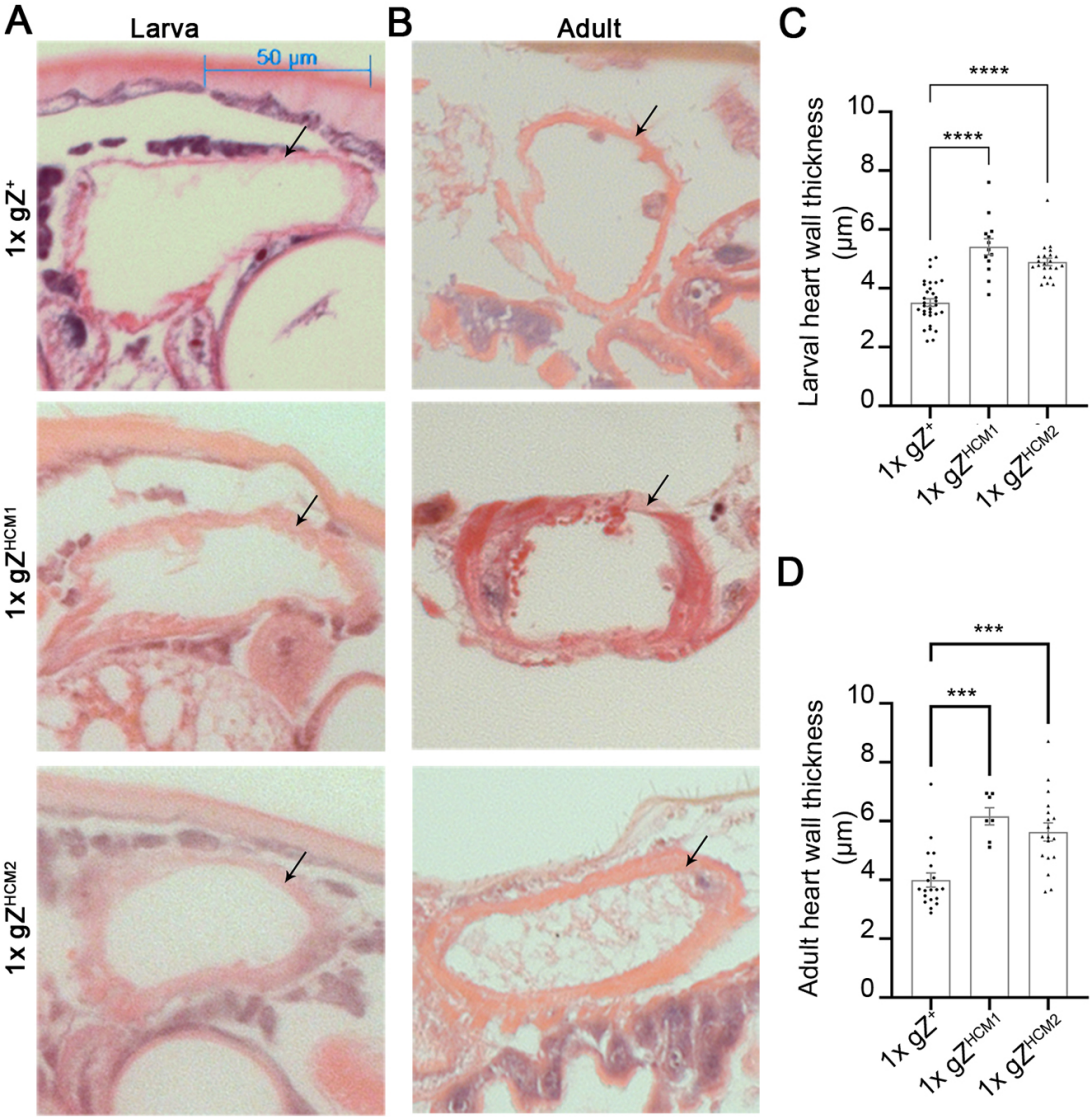
**gZ^HCM^ mutants show markedly increased heart wall thickness**. (A,B) Histological sections in transverse orientations showing heart wall thicknesses of control 1xgZ^+^ (Z^24^;gZ^+^/+) and mutant animals 1xgZ^HCM1^ (Z^24^;gZ^HCM1^/+) and 1xgZ^HCM2^ (Z^24^;gZ^HCM2^/+). In (A), A6/A7 abdominal segments of the heart of 3rd instar larvae. In (B), A1/A2 abdominal segments of the heart of young adults (6-9 days after eclosion). Arrows point at the heart wall. (C,D) Quantification of heart wall thicknesses measured from serial transverse histological sections of larvae 1xgZ^+^ (*n*=32), 1xgZ^HCM1^ (*n*=13) and 1xgZ^HCM2^ (*n*=22) (C), and adult flies 1xgZ^+^ (*n*=15), 1xgZ^HCM1^ (*n*=8) and 1x gZ^HCM2^ (*n*=7) (D). ***P<0.001 and ****P<0.0001 (one-way ANOVA followed by Dunnett's multiple comparison's test). Error bars indicate the mean±s.e.m.

The majority of patients carrying CM-linked ELAC2 variants display dramatic thickening of the left ventricular heart wall soon after birth (Haack et al., 2013; Shinwari et al., 2017). To study the effect of HCM mutations on the morphology of the fly heart, we conducted a histological analysis of hearts obtained from larvae and young adults. We measured the thickness of the heart wall using 5-μm sections of the dorsal vessel sliced in transverse orientation. In general, the heart wall thickness was irregular throughout the circumference of the heart tube (Fig. 5A,B). To account for that, dorsal, ventral and lateral heart wall measurements were collected, and averaged from three consecutive sections. We found that the heart wall thickness of larvae was increased by 54% in 1xgZ^HCM1^ and 39% in 1xgZ^HCM2^ compared to control larvae (Fig. 5C). The effect of mutations on heart wall thickness was more pronounced in adult flies, with an increase of 123% in 1xgZ^HCM1^ and 111% in 1xgZ^HCM2^ (Fig. 5D). These results indicate that HCM mutations lead to heart wall hypertrophy in flies.

### gZ^HCM1^ and gZ^HCM2^ mutations compromise heart function in *Drosophila*

**Fig. 6.**
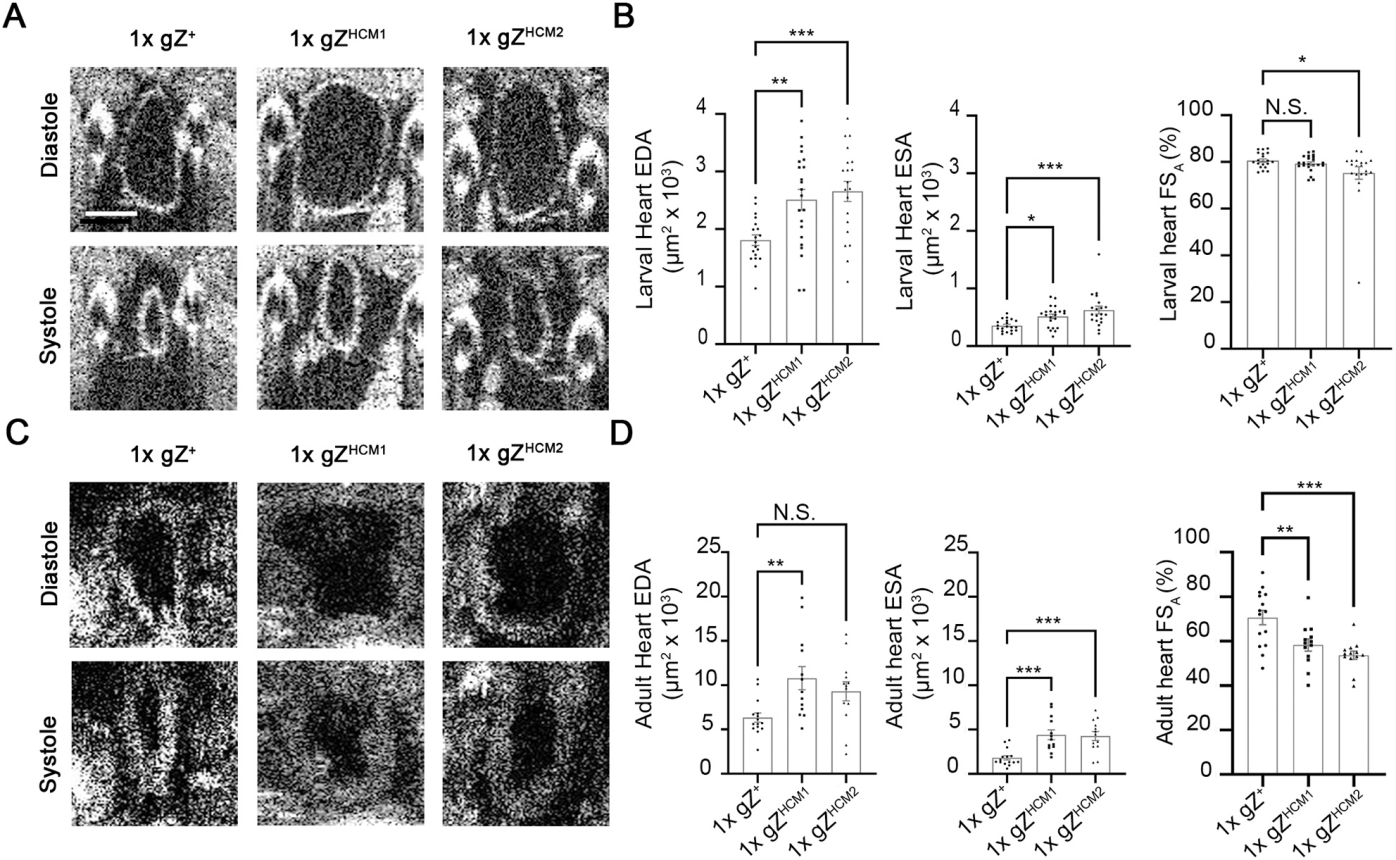
**gZ^HCM^ mutants exhibit dilation of the heart.** (A) OCM images of diastole and systole in A6/A7 heart segment of control 1xgZ^+^ (Z^24^;gZ^+^/+) and mutant larvae – 1xgZ^HCM1^ (Z^24^;gZ^HCM1^/+) and 1xgZ^HCM2^ (Z^24^;gZ^HCM2^/+). Scale bar: 100 µm. (B) Functional analysis of larval hearts. End-diastolic area (EDA), end-systolic area (ESA) and fractional shortening (FS) are shown for 1xgZ^+^ (*n*=19), 1xgZ^HCM1^ (*n*=20) and 1xgZ^HCM2^ (*n*=22) larvae. (C) OCM images of diastole and systole in A1/A2 heart segments of control 1xgZ^+^ (Z^24^;gZ^+^/+) and mutant – 1xgZ^HCM1^ (Z^24^;gZ^HCM1^/+), 1xgZ^HCM2^ (Z^24^;gZ^HCM2^/+) young adult flies. All genotypes were studied 6-9 days after eclosion. (D) Functional analysis of adult fly hearts. EDA, ESA and FS are shown for 1xgZ^+^ (*n*=15), 1xgZ^HCM1^ (*n*=13) and 1xgZ^HCM2^ (*n*=13) flies. N.S., no statistical significance; *P<0.05, **P<0.025, ***P<0.01 (one-way ANOVA followed by Dunnett's multiple comparisons test). Error bars indicate the mean±s.e.m.

Patients with hypertrophic hearts display abnormal ventrical volume and reduced cardiac contractility. Therefore, we investigated whether HCM mutations affect heart activity in larvae and adult flies. We used a high-resolution optical coherence microscopy (OCM) system to image hearts for functional studies. OCM is a non-invasive imaging technology that enables visualization of organs *in vivo* (Alex et al., 2005; Men et al., 2016; Wolf et al., 2006). Light penetrates cuticle and is reflected from the structures underneath, yielding images similar to those obtained when using ultrasound. Representative images of larval and adult heart obtained by OCM are shown in Fig. 6. End-diastolic area (EDA) or end-systolic area (ESA) of the heart lumen when the heart was fully relaxed or contracted, respectively, were measured in transverse orientation.

We found that EDA was increased by 39% in 1xgZ^HCM1^ and 47% in 1xgZ^HCM2^ larvae (Fig. 6C). ESA was also increased by 46% in 1xgZ^HCM1^ and 78% in 1xgZ^HCM2^ compared to age-matched control larvae (Fig. 6C; Movies 1-3).

In adults, hearts of gZ^HCM^ flies showed a diastolic and systolic increase of the lumen area by 111% for 1xgZ^HCM1^ and 107% for 1xgZ^HCM2^ (diastole), and by 176% for 1xgZ^HCM1^ and 206% for 1xgZ^HCM2^ (systole) (Fig. 6D; Movies 4-6).

To summarize, the hearts of gZ^HCM^ flies are dilated at diastole and do not contract efficiently at systole. From these parameters, we calculated fractional shortening (FS) as a measure of heart-tube contractility. In larvae, FS was either the same as in controls (see 1xgZ^HCM1^ in Fig. 6C) or slightly reduced by 6% in 1xgZ^HCM2^ (Fig. 6C). Adult hearts showed a stronger reduction of heart-tube contractility, i.e. a decrease by 17% in 1xgZ^HCM1^ and by 23% in 1xgZ^HCM2^ (Fig. 6D). These data demonstrate that the cardiac function was compromised in flies carrying the HCM-linked sequence variant of RNaseZ.

### gZ^HCM1^ and gZ^HCM2^ mutations lead to an increase in nuclei number and ploidy in *Drosophila* hearts

Heart wall thickness could increase due to cardiomyocyte growth (hypertrophy), proliferation (hyperplasia) or both. Yet, neither of these HCM features were looked at in human patients carrying ELAC2 variants. The *Drosophila* model is very much suitable to study both myocyte hypertrophy and hyperplasia due to the simplicity of the fly heart.

**Fig. 7.**
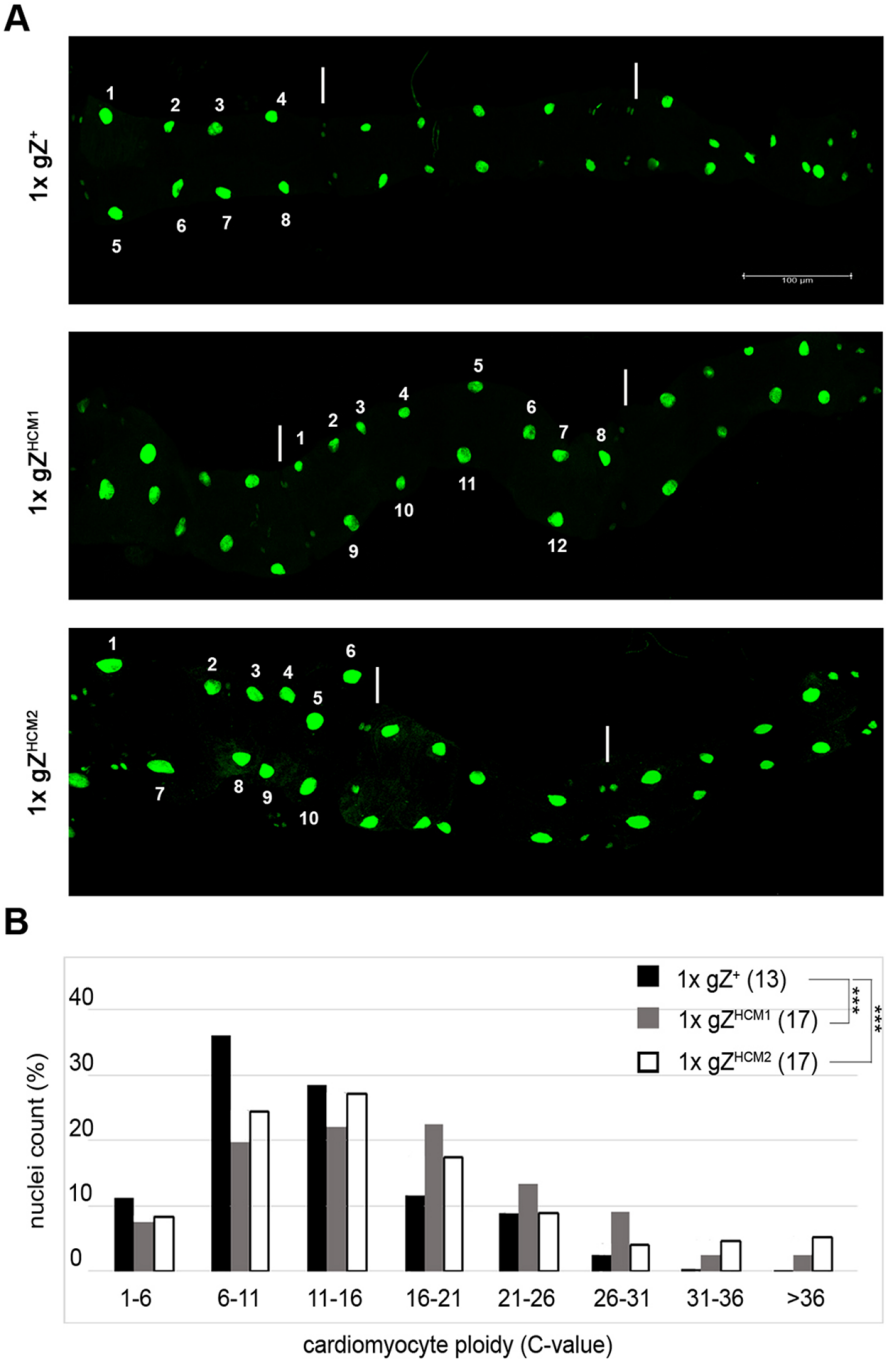
**gZ^HCM^ mutants show an increase in cardiomyocyte nuclei number and ploidy.** (A) Fluorescence imaging of nuclear GFP in hearts of control 1xgZ^+^ (Z^24^;gZ^+^/4xtinC^Δ4^-GFP^NLS^) and mutant young adults – 1xgZ^HCM1^ (Z^24^;gZ^HCM1^/4xtinC^Δ4^-GFP^NLS^) and 1xgZ^HCM2^ (Z^24^;gZ^HCM2^/4xtinC^Δ4^-GFP^NLS^). Flies of all genotypes were studied 1-2 days after eclosion. The A2-A4 abdominal segments are shown; white vertical bars mark the segment boundaries. Scale bar: 100 µm. (B) Plotted is the distribution of cardiomyocyte nuclei with different ploidy (C value) from 1xgZ^+^ (*n*=400 nuclei), 1xgZ^HCM1^ (*n*=534 nuclei), and 1xgZ^HCM2^ (*n*=429 nuclei) as black, gray and white bars, respectively. For each ploidy range, the nuclei number is provided as the percentage of nuclei of total number being studied for each genotype. The mean ploidy C value is shown for each group in parenthesis. ***P<0.01 (one-way ANOVA followed by Dunnett's multiple comparison's test).

To identify and track cardiomyocytes, we engineered a transgenic *Drosophila* strain that expresses GFP in cardiomyocytes, i.e. for a high level of cardiac-specific expression, we cloned GFP ORF under promoter featuring four tandem repeats of the tinC^Δ4^ cardiac enhancer from the *Drosophila tinman* gene (Lo and Frasch, 2001). The resulting transgene 4xtinC^Δ4^-GFP^NLS^ allowed clear visualization of the nuclei of all cells within the heart, including myocytes, the heart valve and ostia cells, at every developmental stage. To quantify cardiac nuclei under normal and hypertrophic conditions, we introduced the 4xtinC^Δ4^-GFP^NLS^ transgene into *RNaseZ* wild-type (Z^24^;gZ^+^/4xtinC^Δ4^-GFP^NLS^) and mutant (Z^24^;gZ^HCM^/4xtinC^Δ4^-GFP^NLS^) genetic backgrounds. First, we confirmed that wild-type control flies consistently displayed eight nuclei per segment (*n*=19) (Fig. 7A). Amazingly, we found more nuclei per heart segment in mutants – ≤13 for 1xgZ^HCM1^ and ≤10 for 1xgZ^HCM2^ (Fig. 7A). These extra cardiomyocyte nuclei were found in 55% of 1xgZ^HCM1^ (*n*=21) and 38% of 1xgZ^HCM2^ (*n*=18) flies examined.

In general, cell size is proportional to the amount of nuclear DNA (Edgar and Orr-Weaver, 2001). Many post-mitotic cells, e.g. mammalian cardiac myocytes, grow by endoreplication, a process of multiple rounds of genomic DNA replication without cytokinesis (Ahuja et al., 2007). Thus, to evaluate potential cardiomyocyte hypertrophy in gZ^HCM^ flies, we measured the ploidy of heart cells. Consistent with previous studies (Yu et al., 2013), our DAPI staining showed varied ploidy of wild-type *Drosophila* cardiomyocytes, ranging from four to 36, with a mean C value of 13 (Fig. 7B). Importantly, mutant cardiomyocytes showed increased ploidy – the mean C value for 1xgZ^HCM1^ and 1xgZ^HCM2^ was 17, *P*<0.001 (Fig. 6B). Our data clearly demonstrate that both RNaseZ variants yielded hypertrophic hearts built of cardiomyocytes with increased ploidy and, probably, increased size.

### gZ^HCM1^ and gZ^HCM2^ mutations lead to increased deposition of ECM components in *Drosophila* hearts

*Drosophila* heart is embedded in extracellular matrix (ECM), the components of which are secreted by pericardial cells, cardiomyocytes and adipocytes (Rotstein and Paululat, 2016; Vaughan et al., 2018). In mammals, myocardial fibrosis (increased amounts of ECM proteins) is a hallmark of HCM and correlates with the degree of hypertrophy, impaired ventricular performance and diastolic dysfunction (Ho et al., 2010; Teekakirikul et al., 2012). In *Drosophila*, deposition of extra amounts of collagen and collagen-like proteins is also tied to decreased cardiac performance and hypertrophy (Na et al., 2013; Vaughan et al., 2018). Therefore, we explored whether fibrosis is part of the phenotype associated with gZ^HCM^ mutations.

**Fig. 8.**
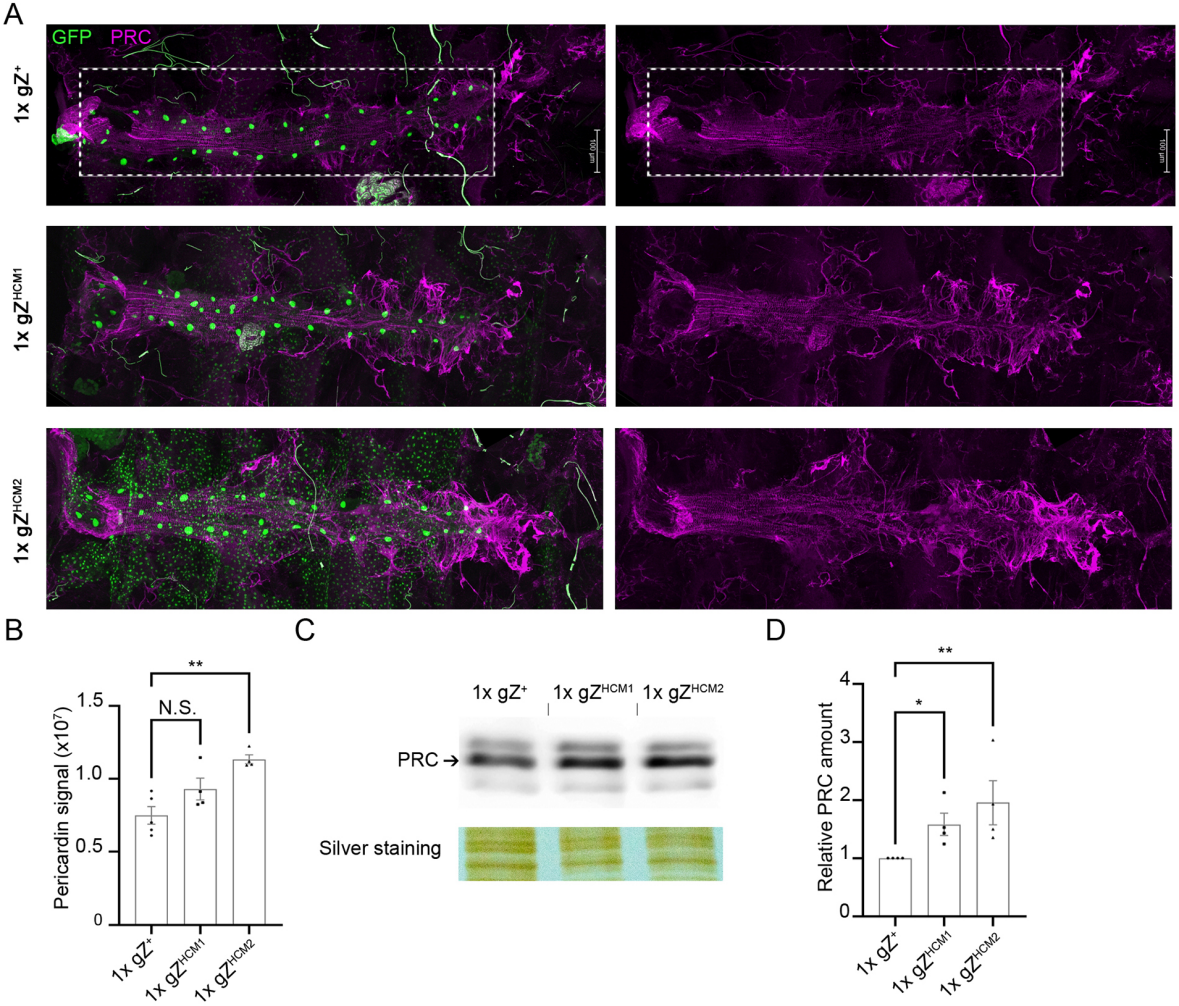
**gZ^HCM^ mutants show an increased deposition of pericardin.** (A) Representative confocal images of hearts from control 1xgZ^+^ (Z^24^;gZ^+^/4xtinC^Δ4^-GFP^NLS^) and mutant young adults – 1xgZ^HCM1^ (Z^24^;gZ^HCM1^/4xtinC^Δ4^-GFP^NLS^) and 1xgZ^HCM2^ (Z^24^;gZ^HCM2^/4xtinC^Δ4^-GFP^NLS^) – stained against pericardin (PRC). All fluorescent images were taken by using the same exposure parameters. Fluorescent intensities were measured within the region of interest selected over A1-A4 segments of the heart tube (boxed areas). Flies of all genotypes were studied 1-2 days after eclosion. (B) Average pixel intensity of PRC immunostaining, *n*=4. (C) Western blot of total proteins from isolated hearts of control (1xgZ^+^) and mutant flies (1xgZ^HCM1^ and 1xgZ^HCM2^) stained against PRC. Silver staining was used as a loading control. (D) Quantification of PRC levels (arbitrary unit) relative to loading control, normalized to the control genotype. Data are the average of four independent experiments. N.S., not statistically significant; *P<0.05, **P<0.025, one-way ANOVA followed by Dunnett's multiple comparison's test. Error bars, mean±s.e.m.

We specifically focused on accumulation of pericardin (PRC), an insect collagen IV-like ECM protein found exclusively around the dorsal vessel. Immunostaining of dissected hearts from 2-day-old mutant flies for PRC showed a quantifiable increase in the PRC immunofluorescence signal compared to that in hearts from age-matched control flies (Fig. 8A,B). Western blot analysis of total protein from isolated hearts confirmed increased levels of PRC in gZ^HCM^ mutants compared to those of control hearts (Fig. 8C,D). Thus, it appears that fibrosis also attributes to a hypertrophic heart in gZ^HCM^ flies.

## DISCUSSION

### Partial functional homology of ELAC2 and RNaseZ

Generation of functional tRNA 3′-ends is essential for all organisms. Eukaryotes universally employ highly conserved endoribonucleases for this purpose. Both human ELAC2 and fly RNaseZ proteins possess the same biochemical activity as they cleave off the tRNA 3′-end *in vitro* and *in vivo* (Dubrovsky et al., 2004; Takaku et al., 2003). We, therefore, investigated the extent of biological conservation of function of human and *Drosophila* enzymes *in vivo*. We produced flies carrying the hs-*ELAC2* transgene in an *RNaseZ* null background. Rescue experiments revealed that the human homolog was only partially substituting for its *Drosophila* counterpart. Human ELAC2 complements the function of *Drosophila* RNaseZ at larval stages, leading to larval growth, molting and even pupariation of some of the animals. However, most of the rescued animals stall their developmental progress during fully grown 3rd instar stage and die 20 days later. Only a small number (59 out of 240) of rescued larvae pupariated at approximately day 9 AED; however, their development did not progress much further and they died as early pupae. Thus, it appears that, even though human and *Drosophila* lineages separated more than 700 million years ago (Hedges et al., 2006), two proteins retained some functional homology. Nonetheless, the inability of ELAC2 to rescue RNaseZ KO flies into adulthood, clearly reflects the divergence of their biological functions.

Apparently, RNaseZ has some functions not carried out by ELAC2. This might be due to the fact that humans have two forms of this protein – the long ELAC2 (RNaseZ^L^) and the short ELAC1 (RNaseZ^S^) version – whereas only a long version exists in *Drosophila*. ELAC2 has been shown to colocalize with nuclei and mitochondria, whereas ELAC1 resides in the cytosol (Takahashi et al., 2008). Until recently, only little was known about ELAC1 function but a new study has shown that ELAC1 has a role in tRNA recycling, which takes place in cytosol and, thus, cannot be completed by ELAC2 (Yip et al., 2020). It appears that in flies, RNAseZ carries out functions that are in humans performed by both ELAC1 and ELAC2. In *Drosophila* cells, RNaseZ has been detected in all three compartments – nuclei, mitochondria and cytosol, making it possible for the *Drosophila* protein to have some activities similar to ELAC1. In addition, RNaseZ might have some other – yet unknown – roles in flies. Consequently, replacing RNaseZ with ELAC2 may not restore all of the essential functions of RNaseZ in flies, which is a likely explanation for the incomplete rescue. It would be interesting to see whether supplying both human ELAC1 and ELAC2 can rescue an RNaseZ KO in flies.

Thus, we conclude that, even though ELAC2 and RNaseZ appear to be highly conserved, their functions have diverged.

### F154L and T520I ELAC2 alleles are causally connected to CM

In humans, multiple mutations of *ELAC2* have been associated with the early onset of cardiac hypertrophy and decompensation (Haack et al., 2013; Shinwari et al., 2017), although the causal connection between mutant ELAC2 forms and CM has not been explicitly established. In our study, we used RNaseZ – a *Drosophila* homolog of ELAC2 – to generate fly models carrying human CM-associated alleles. Our histological analysis established that flies with mutant RNaseZ had hypertrophic heart wall (Fig. 5). Moreover, using OCM technology we found that the size of the heart lumen, as assessed at both diastole and systole, was increased (Fig. 6C,D). Finally, judging by FS (Fig. 6C,D) the contractility of the heart was reduced. Notably, the early onset of the CM phenotype in *Drosophila* was consistent with the early appearance of CM symptoms in patients carrying ELAC2 variants. Thus, our data provided direct experimental evidence that the F154L and T520I mutations of ELAC2 are the primary cause of this heart pathology.

Interestingly, we found that characteristic traits of heart impairment are already apparent during the larval phase of development but they became much stronger and fully pronounced in adult gZ^HCM^ flies. *Drosophila* heart develops early in embryogenesis; during larval stages and metamorphosis it undergoes further growth and differentiation but without cell proliferation. We did not expect any heart phenotype in embryos, as the *RNaseZ* gene has a maternal effect, i.e. its mRNA is produced and deposited into the oocyte during oogenesis (Xie et al., 2013). As soon as maternal RNaseZ stores are depleted, cardiomyocytes start to experience the damaging effect of RNaseZ variants. Given that RNaseZ processes both mitochondrial and nuclear pre-tRNA transcripts, its activity is required throughout animal development (Dubrovsky et al., 2004; Xie et al., 2013). This explains why adults display the strongest phenotype – cardiomyocytes accumulate the damage produced by RNaseZ mutant variants from larva-to-pupa-to-adult.

It appears that the hearts of gZ^HCM^ flies combine features of both hypertrophic and dilated cardiomyopathy, reminiscent of the dilated stage of hypertrophic cardiomyopathy (D-HCM) reported for some patients with ELAC2 mutations. Whereas dilated and hypertrophic cardiomyopathies are traditionally classified as two distinct pathologies, it has been suggested that, in some cases, they represent consecutive stages in the progression of the same disease (Bingisser et al., 1994; Harris et al., 2006; Hamada et al., 2010). Having a fly model with the features of a rare form of cardiac disease, offers an opportunity to study the underlying mechanisms.

Some studies that describe CM cases associated with ELAC2, have proposed incomplete processing of mitochondrial transcripts as the cause of organelle malfunction that, in turn, leads to heart hypertrophy (Haack et al., 2013; Saoura et al., 2019). The fly model established by us and described in this study will be extremely useful in testing this hypothesis.

### Increased ploidy of cardiomyocytes and accumulation of ECM components are aspects of the cardiac hypertrophy phenotype in adult flies

In general, heart wall enlargement could result from either an increase in cardiomyocyte number (hyperplasia) and/or size (hypertrophy), accumulation of ECM components (fibrosis), or a combination of these processes. Hypertrophy and fibrosis are well-known hallmark features of HCM in humans and flies (McKenna et al., 1981; Marian and Braunwald, 2017; Na et al., 2013); however, hyperplasia has not been detected and/or studied, except for the few cases in the mouse HCM model (Becker et al., 2011; Farrell et al., 2017). As to HCM patients carrying ELAC2 variants, none has been tested for hyperplasia, hypertrophy or fibrosis.

The heart tube of adult *Drosophila* comprises 84 cells, all of which originate during embryogenesis from cardiac progenitor cells (Rotstein and Paululat, 2016). While proceeding through the larval stages, the size of the heart tube increases, although this enlargement does not involve myocyte proliferation but, rather, cell growth by endoreplication (also known as endocycling) – a specialized cell cycle during which cells increase their ploidy and size without karyokinesis or cytokinesis (Edgar and Orr-Weaver, 2001; Edgar and Nijhout, 2004; Zielke et al., 2013). Thus, the heart of the adult fly is entirely postmitotic and composed of polyploid mononucleated cardiomyocytes (Yu et al., 2013; this study). By using RNA interference (RNAi) and genetic knockout, we have previously shown that *Drosophila* RNaseZ is required for cell growth via endoreplication but that its absence does not directly affect DNA replication (Xie et al., 2011, 2013). In this current study, we tested two hypomorphic alleles – gZ^HCM1^ and gZ^HCM2^ – and found that both cause an increase in cardiomyocyte ploidy by ∼32% on average (Fig. 7B). Moreover, our data showed that flies with HCM-linked sequence variants of RNaseZ develop hearts with more cardiomyocyte nuclei per segment than those in wild-type fly (Fig. 7A). This phenomenon illustrates that cardiomyocytes of gZ^HCM^ mutant flies not only replicate DNA but, after the replication step, may enter mitosis, ending with either cytokinesis (resulting in hyperplasia) or karyokinesis (resulting in polyploid binucleated cells).

It is not clear yet what prompts cardiomyocytes of flies that carry gZ^HCM^ alleles to re-enter and go through additional rounds of endocycling. Previously, we have shown that loss of mitochondrial RNaseZ activity increases levels of intracellular reactive oxygen species (ROS), resulting in genotoxic stress (Xie and Dubrovsky, 2015). A recent study proposed that postmitotic cells experiencing oxidative stress and associated DNA damage may gain protection by initiating endoreplication and developing polyploidy (Nandakumar et al., 2020). Therefore, one potential pathway that connects *RNaseZ* mutant alleles and heart hypertrophy could be mediated by ROS, although further experiments are required to confirm this suggestion.

Given that fibrosis is a well-documented characteristic of HCM, we studied the ECM in gZ^HCM^ flies by looking at levels of PRC, a prominent ECM protein deposited exclusively around the *Drosophila* heart tube. Notably, flies carrying gZ^HCM1^ and/or gZ^HCM2^ alleles displayed higher levels of PRC compared to control flies (Fig. 8). Based on investigations published by others (Na et al., 2013; Vaughan et al., 2018), PRC accumulation could be one of the reasons of heart malfunction in gZ^HCM^ flies.

Overall, our current study not only provides the proof that directly implicates ELAC2 sequence variants in HCM, but offers a convenient model to study mechanisms and pathway leading to heart pathology.

### HCM-linked mutations decrease longevity and physical fitness in flies

In addition to studying the structure and function of the heart, we also examined our HCM fly model for other traits that could be reflective of symptoms displayed by patients with cardiomyopathy. We noticed that HCM flies experienced delayed development, similar to intra-uterine growth retardation in humans. We also found that RNaseZ knockout flies carrying gZ^HCM^ transgenes have a dramatically shortened lifespan, which is reminiscent of the decreased life expectancy of HCM patients (Akawi et al., 2016; Haack et al., 2013; Kim et al., 2017; Shinwari et al., 2017). Lastly, by using the negative geotaxis climbing assay, we observed that Z^HCM1^ and Z^HCM2^ mutations decrease fly fitness levels. This feature is consistent with muscle hypotonia and retarded psychomotor development in human patients (Akawi et al., 2016; Haack et al., 2013; Kim et al., 2017; Shinwari et al., 2017).

Overall, gZ^HCM^ flies exhibit multiple phenotypes that are similar to symptoms in humans. In both organisms, HCM-linked mutations affect heart morphology and function, fitness level, rate of development and longevity.

### Comparison of mutant *RNaseZ* transgenes

Based on our analysis of gZ^HCM1^ and gZ^HCM2^ alleles, the latter has a stronger negative effect on all traits that were studied by us – longevity, level fitness and heart function. Importantly, these observations are consistent with those in humans. For example, patients with the T520I allele (gZ^HCM2^) live – at most – to the age of 6 months, whereas patients with the F154L variant (gZ^HCM1^) may live up to 13 months of age. This difference could be explained by the fact that the T520I mutation affects a highly conserved residue in motif I. The *in silico* analysis of the ELAC2/RNaseZ structure shows that motif I interacts with motifs II-V, the latter of which together form the catalytic center (Saoura et al., 2019). This suggests that even subtle changes of motif I, such as loss of polarity due to T520I substitution, potentially impairs the efficacy of catalysis.

The gZ^HCM1^ mutation is located in the N-terminal half of the RNaseZ protein, between pseudo motifs II and III. Based on the tertiary structure of ELAC2/RNaseZ, the site of gZ^HCM1^ (F154L) is positioned close to the interface between the N- and C-terminal MBL domains, approaching the motif II region of the C-terminal half (Saoura et al., 2019). It has been suggested that reducing the size of the hydrophobic side chain, such as the case of F154L substitution, affects the folding of the region, which is crucial for binding metal ions and efficient catalysis (Saoura et al., 2019).

Although both mutations potentially affect catalytic activity of ELAC2/RNaseZ, the gZ^HCM2^ position is located within a highly conserved region, implying that this missense mutation can bring upon a stronger damage to the function of the enzyme than gZ^HCM1^. This explains a more-pronounced negative effect of this mutation both in humans and in flies.

## MATERIALS AND METHODS

### Fly stocks

Flies were maintained at 25°C on standard cornmeal-molasses-agar medium. The *RNaseZ* knockout allele used in this study (Z^24^) as well as the hs-RNZ-V5 (hs-Z^+^) and genRNZ-V5 (gZ^+^) constructs and corresponding transgenic flies are described elsewhere (Xie et al., 2011, 2013).

### DNA cloning and generation of transgenic fly strains

To generate the hs-ELAC2-V5 (hs-ELAC2) construct, the ELAC2 coding sequence was joined at the C-terminal end with the V5-epitope sequence and placed under the heat-inducible promoter in the pCaSpeR-hs vector. Transgenic flies were generated by P element-mediated germline transformation. Several homozygous viable transgenic lines carrying this construct on the third chromosome were established. Transgene expression was activated by heatshock (HS) treatment at 37°C for 1 h; full-size protein synthesis was confirmed by western blot hybridization with the anti-V5 antibody (Fig. S1).

To introduce CM-linked mutations into RNaseZ we amplified fragments of *RNaseZ* with the following pairs of primers: (5′-AATTGCCTCGGCCAAGGATC-3′ with 5′-CACCACGA**G**ACGTCGCATTGAC-3′ and 5′-CGACGT**C**TCGTGGTGCTAAAGAATCTC-3′ with 5′-AGCAGAATAAGTGGGTCTGCC-3′) for Z^HCM1^, (5′-AATTGCCTCGGCCAAGGATC-3′ with 5′-GACCATAA**A**TTCCTTCTCCACAATCC-3′ and 5′-GTGGAGAAGGAA**T**TTATGGTCAAATTG-3′ with 5′-AGCAGAATAAGTGGGTCTGCC-3′) for Z^HCM2^ (letters in bold indicate introduced nucleotide changes). We used NEBuilder HiFi DNA Assembly master mix (New England BioLabs) to insert the amplified fragments into BamHI and AvrII sites of the genRNZ-V5 construct (Xie et al., 2011), yielding the genRNZ^HCM1^-V5 (gZ^HCM1^) and genRNZ^HCM2^-V5 (gZ^HCM2^) constructs. *E. coli* transformants were picked, and successful cloning was confirmed by restriction digestion and sequencing. To establish transgenic lines, we used phiC31-mediated site-specific integration to insert the transgenes into the AttP site on third chromosome at 68A4 (Groth et al., 2004).

To create a strong heart-specific promoter, the 303 bp tinC^Δ4^ enhancer element of the *tinman* gene was amplified with the following pairs of primers (5′-ctatagggcgaattgggtacctctagaCATGAACAGCTTTCGATCG-3′ with 5′-AaggaatccctAAGCGGAAATTGTGGTGTTTTC-3′ and 5′-atttccgcttAGGGATTCCTGGGGAGGG-3′ with 5′-aaaagctggagctccaccgcggcctaggtGGAGGCAGGGAAACATTTTAC-3′), where capital letters indicate the gene-specific part in each primer (Lo and Frasch, 2001). To generate the 4xtinC^Δ4^ fragment we used the NEBuilder HiFi DNA Assembly master mix to sequentially introduce four tandem insertions of tinC^Δ4^ into SacII and KpnI sites of the pBlueScriptII plasmid. After confirming the integrity of the 4xtinC^Δ4^ fragment by DNA sequencing, it was cut out, purified and ligated into XbaI and SacII sites of pH-Stinger vector (DGRC), yielding the 4xtinC^Δ4^-GFP^NLS^ construct. Transgenic flies were generated by P element-mediated germline transformation. Several homozygous viable transgenic lines carrying this construct on 3rd chromosome were established. Transgene expression was confirmed by GFP accumulation in cardiomyocyte nuclei.

### ELAC2 rescue analysis

For ELAC2 rescue experiments we used offspring of the Z^24^/CyO,GFP;hs-ELAC2 line. In the same experiment, offspring of Z^24^/CyO,GFP served as a negative control and offspring of Z^24^/CyO,GFP;hs-Z^+^ as positive control. Larvae homozygous for the Z^24^ allele were selected by the absence of the GFP marker. All animals were subjected to HS treatment for 1 h, every 24 h from day 1 after egg deposition (AED).

### Western blot analysis

For adult fly analysis, protein extracts were made by homogenizing five whole animals in Laemmli sample buffer. For heart analysis, ten hearts were dissected from 1- to 2-day-old adult female flies, homogenized in 25 µl ECM extraction buffer (1 mM EDTA, 1,5% Triton X-100 and 2 M urea) and then supplemented with 25 µl 2× Laemmli buffer. All samples were boiled at 99°C for 3 min. Aliquots were separated on a SDS-polyacrylamide gel and subjected to either western blotting or silver staining. For PRC analysis, blotting buffer was supplemented with 2 M urea. Blots were hybridized overnight at 4°C with primary antibodies diluted in PBST supplemented with 5% dry milk powder (w/v). Antibodies used were anti-V5 (Invitrogen, 1:10,000), anti-α-tubulin (Sigma, 1:5000) and anti-pericardin (PRC, DSHB, 0.2 µg/ml). Silver staining was performed using SilverXpress staining kit (Life Technologies) following the manufacturer's protocol. Visualization of blots was done using KwikQuant Digital Western Blot Detection System (Kindle Biosciences). Band densities were quantified using the KwikQuant imaging software.

### Longevity assay

To measure the lifespan, flies of indicated genotypes were collected on the day of eclosion and placed into same-sex cohorts of ten flies per vial. The number of survivors in each vial was scored daily. The flies were placed in fresh vials three times per week (Monday, Wednesday and Friday) during the entire test. Statistical analysis was performed using Mantel–Cox test.

### Negative geotaxis climbing assay

Locomotor activity of the flies was tested using the geotaxis assay described elsewhere (Gargano et al., 2005; Piazza et al., 2009). Flies were collected on the day of eclosion under brief CO_2_ anesthesia (1-2 min), sorted in groups of ten (five males and five females) and allowed to recover at least 18 h at 25°C prior to the assay. Next day, flies were transferred into vials marked by lines forming four equally spaced quadrants. Each vial was gently tapped to bring flies to the bottom of the vial and to initiate the negative geotaxis response. Flies were allowed to climb up for 4 s before a photograph was taken to record the position of each fly in the vial. Each fly was assigned a rating based on the quadrant it reached; flies that did not climb and stayed at the bottom were scored as zero. The weighted average of three consecutive trials was calculated for each vial, representing the climbing index for that vial.

### Histological analysis

To ensure all animals are studied at the same developmental point, we synchronized them by behavioral and morphological criteria as described elsewhere (Xie et al., 2011). Procedure to fix and process larvae for histological analysis is described elsewhere (Velentzas and Baehrecke, 2021). Briefly, 3rd instar larvae were fixed in FAAG solution (80% EtOH, 5% acetic acid, 4% formaldehyde, 1% glutaraldehyde) for 24 h at 4°C. The procedure to fix and process adult flies was adapted from previously published protocols (Kucherenko et al., 2010; Yu et al., 2010). Briefly, adult flies (6-9-day-old females) were fixed in Carnoy's fixative overnight at 4°C. After fixation, both larval and adult samples were dehydrated using increasing gradients of EtOH solution, washed with xylenes and then placed into hot paraffin. Solidified paraffin blocks were sliced at transverse orientation at 5 µm thickness; sliced sections were placed on slides for subsequent haematoxylin and eosin (H&E) staining.

To ensure heart wall measurements were consistently taken at the transition of the A1-to-A2 segment in adult flies, we used the following approach: i) we identified sections sliced through the anterior thoracic end; ii) we removed the next 500 µm of tissue, which brought us into abdomen, ∼50 µm from the posterior end of the thorax; iii) we collected three consecutive 5-µm thick sections for analysis. For larvae, heart wall thickness was analyzed at 450-500 µm from the larval posterior end, which corresponds to the A6/A7 segments.

Sections were rehydrated and H&E stained (Sigma). All histological samples were analyzed under a Zeiss Axio Imager M1 microscope, brightfield images were captured with an AxioCam MRc camera. Wall thickness was calculated as the average of three measurements from each section in three consecutive sections of the heart.

### OCM analysis of cardiac function

For optical coherence microscopy (OCM), white light from a supercontinuum source (SC-400-4, Fianium Ltd., UK) with a central wavelength of ∼800 nm and a bandwidth of ∼220 nm was used. We used a 45° rod mirror to generate an annular sample probe beam to obtain an extended depth of focus. The axial and transverse resolutions provided by the OCM system were 1.5 μm and 3.9 μm, respectively. The backscattered light from the reference and sample arms was detected using a spectrometer comprising a 600 lines/mm transmission grating (Wasatch Photonics, Logan, UT, USA) and a 2048 pixel line-scan camera (AViiVA EM4, e2v technologies plc, UK), operated at 20k A-scans/s. Sensitivity of the system was determined to be ∼95 dB with a sample arm power of 5 mW. M-mode images were acquired at a frame rate of 128 Hz.

To ensure consistency of the heart tube location chosen for OCM imaging, we employed an approach proposed and published elsewhere (Men et al., 2016). Briefly, all specimens were imaged first in the longitudinal orientation to identify the cardiac chamber and to adjust the position of an animal, so that the widest area of the heart is in the focal plane of the imaging beam in transverse orientation. Then, animals were imaged in the transverse orientation to center the heart chamber.

Larval hearts were studied in A6/A7 segments. We measured end-diastolic areas (EDAs) and end-systolic areas (ESAs) of 30 consecutive best-visible heartbeats per larva, using ImageJ (National Institutes of Health, USA). Fractional shortening (FS) was calculated as (EDA−ESA)/EDA×100%.

Adult hearts were studied at the transition of A1-to-A2 segment (∼50 µm from the posterior end of the thorax), where the heart chamber is at its widest and best visible. Usually, adult heart contractions display some variability over time. To account for that, we collected EDA and ESA measurements from the heart beats spaced at 3-s intervals throughout the 30 s of time-lapse OCM images, giving us ten impartially selected heart beats from each recording.

### Heart immunostaining analysis

Adult females were collected on day 2 after eclosion, and hearts were dissected but kept on the cuticle as previously described (Vogler and Ocorr, 2009). Briefly, the head and thorax were removed, and the abdomen was placed in the Artificial *Drosophila* Hemolymph (ADH) buffer (5 mM HEPES pH 7.1, 108 mM NaCl, 5 mM KCl, 2 mM CaCl_2_, 8 mM MgCl_2_, 1 mM NaH_2_PO_4_, 4 mM NaHCO_3_, 10 mM sucrose, and 5 mM trehalose). The abdomen was then cut open and internal organs were removed. ADH buffer that contained 10 mM EGTA was added for 1 min to relax the cardiac muscle. Next, samples were fixed in 4% formaldehyde in PBS for 20 min at room temperature. To stain for pericardin (PRC), samples were hybridized overnight at 4°C with monoclonal anti-PRC antibody (DSHB, 2 µg/ml) diluted in PBS supplemented with 0.3% Triton X-100 (PBS/TX) buffer. After three washes (10 min per wash) with PBS/TX, samples were hybridized for 1 h at room temperature with secondary goat anti-mouse antibody conjugated to Alexa Fluor 647 (Jackson Immunoresearch, 1:400) diluted in PBS/TX. Next, cuticles were washed three times (10 min per wash) in PBS/TX and mounted on a glass slide using Vectashield antifade medium (Vector Laboratories) and squashed by the coverslip for 10 s.

Nuclei of cardiomyocyte were identified by the presence of GFP. The stained heart preparations were visualized with a Leica TSP5 laser scanning confocal microscope.

### Cardiomyocyte ploidy analysis

Cardiomyocyte ploidy was determined by adapting methods described elsewhere (Xie et al., 2013; Yu et al., 2013). Adult *Drosophila* were collected on day 2 after eclosion, and hearts were dissected but kept on the cuticle in ADH buffer (see above). Imaginal wing discs from wild-type 3rd instar larvae were also dissected and used as a diploid reference for DNA quantification. For all following steps hearts and wing discs were treated in parallel. Tissues were fixed in 4% formaldehyde/PBS at room temperature for 20 min, washed three times (10 min per wash) in PBS/TX and stained at room temperature in the dark for 10 min with Hoechst 33342 (Molecular Probes) diluted 1:1000 in PBS/TX. Next, samples were washed three times (10 min per wash) in PBS/TX, hearts pulled off the cuticle and placed on a glass slide together with wing discs, mounted with Vectashield antifade medium and squashed by the coverslip for 10 s. The samples were then analyzed by obtaining z-stacks of Hoechst-stained nuclei using Leica TSP5 laser scanning confocal microscope. Cardiomyocytes were identified by the presence of GFP in their nuclei. The intensity of Hoechst staining for each cardiomyocyte and wing disc nuclei was quantified using ImageJ. To assess the ploidy, the staining intensity of the cardiomyocyte nuclei was normalized to the intensity of wing disc nuclei in each independent experiment. Ploidy was expressed as the C-value, where C=1 refers to the amount of DNA within a haploid nucleus.

### Statistical analysis

All graphs and statistical analyses were performed in Microsoft Excel and GraphPad Prism9. Statistical data are presented as the mean±s.e.m. *P* values for all the comparisons were determined by one-way ANOVA followed by Dunnett's multiple comparison's test unless specified otherwise. The mean difference was considered statistically significant at the 95% confidence level. Results were considered as not significant at *P*>0.05, significant *P<0.05, very significant **P<0.025 and extremely significant ***P<0.001. Figures were assembled with Adobe Photoshop (Adobe Systems, San Jose, CA). Sequence alignment was performed with Clustal Omega 2 (Madeira et al., 2019).

## Supplementary Material

Supplementary information
