# Supplementary material for: ELAC2/RNaseZ-linked cardiac hypertrophy in Drosophila melanogaster
Source: Dis Model Mech. 2021 Aug 31;14(8):dmm048931. doi: 10.1242/dmm.048931 (PMC8419712; doi:10.1242/dmm.048931)
Supplement: Supplementary information [file dmm-14-048931-s1.pdf]

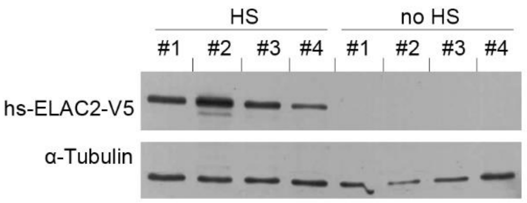

Fig. S1. hs-ELAC2-V5 construct expression. Western blot analysis of the V5-tagged hs-ELAC2 constructs after heat shock (HS) or no HS treatments. α-Tubulin is a loading control.

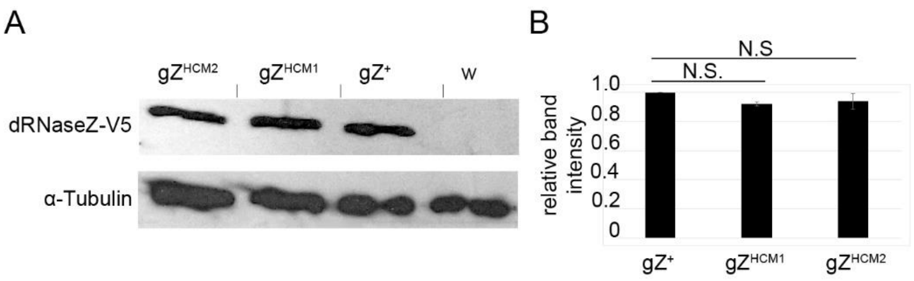

Fig. S2. The level of dRNaseZ protein expression was not affected by CM-linked mutations. **A.** Western blot analysis of the V5-tagged variants of dRNaseZ protein. w is white[118] genotype used as a negative control. α-Tubulin is a loading control. **B.** Data shown is dRNaseZ-V5 band intensity relative to the loading control (α-Tubulin). Data are the average of measurements for each genotype from three independent experiments. N.S. – not statistically significant, two-tail t-test Error bars, mean ± s.e.m.

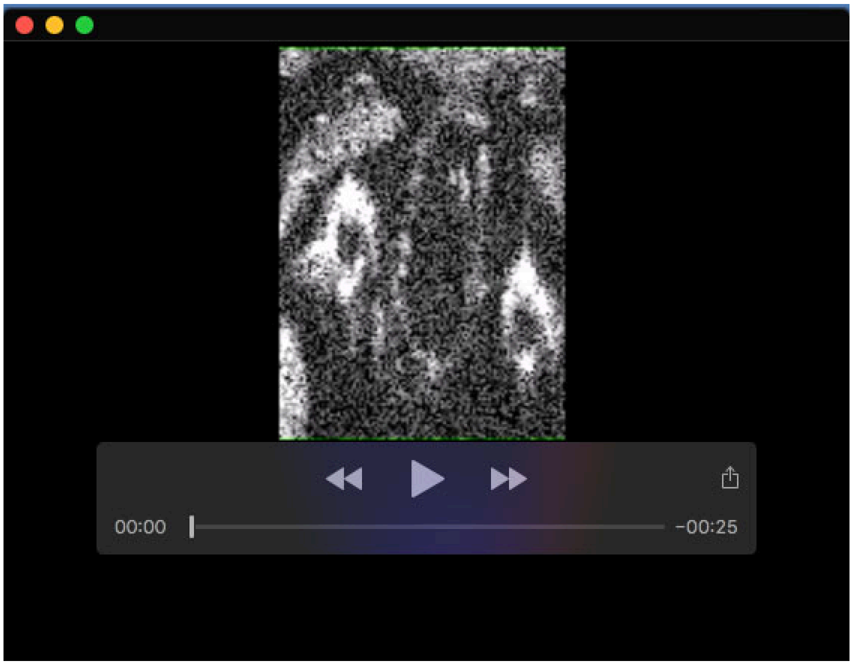

Movie 1. M-mode video of Z^{24}; gZ^{+}/+ beating heart in 3rd instar. Video is captured around A7 segment.

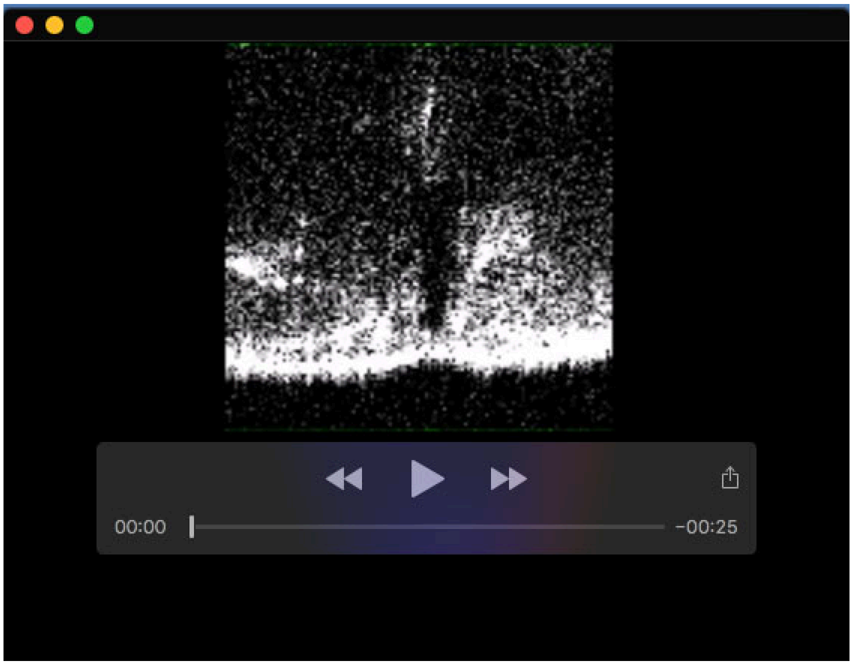

Movie 2. M-mode video of Z^{24}; gZ^{HCM1}/+ beating heart in 3rd instar. Video is captured around A7 segment.

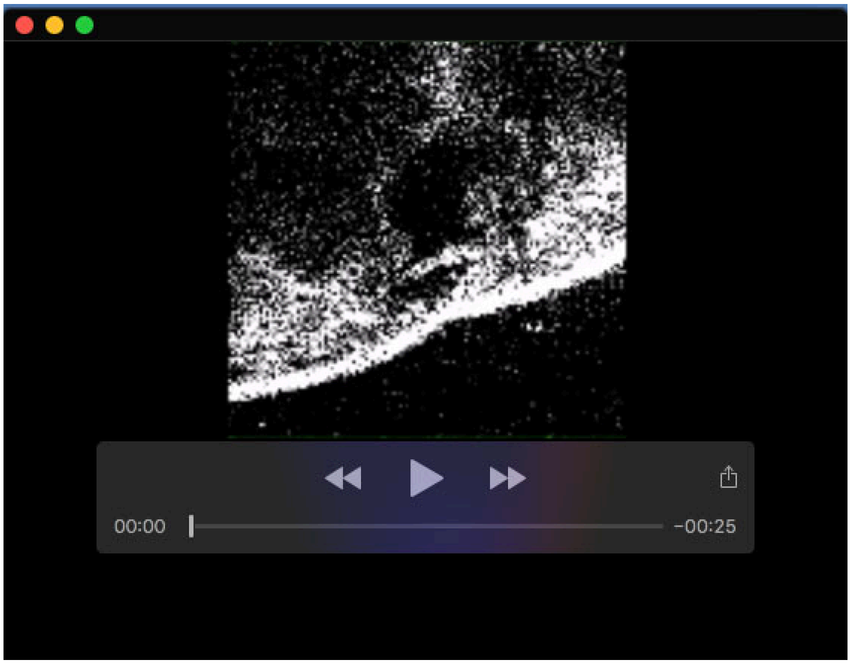

Movie 3. M-mode video of Z^{24}; gZ^{HCM2}/+ beating heart in 3rd instar. Video is captured around A7 segment.

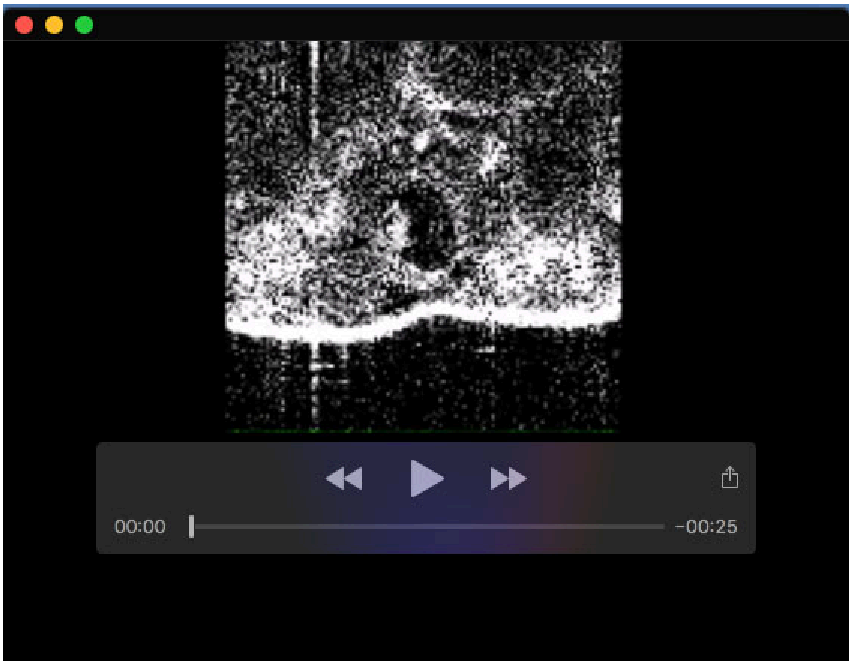

Movie 4. M-mode video of 9 day old Z^{24}; gZ^{+}/+ beating adult heart. Video is captured around A1 segment.

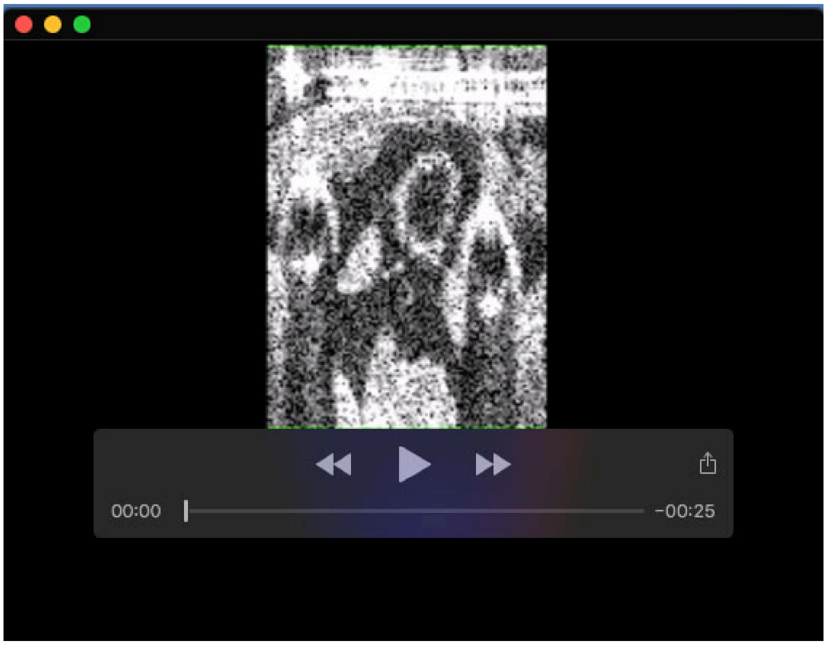

Movie 5. M-mode video of 9 day old Z^{24}; gZ^{HCM1}/+ beating adult heart. Video is captured around A1 segment.

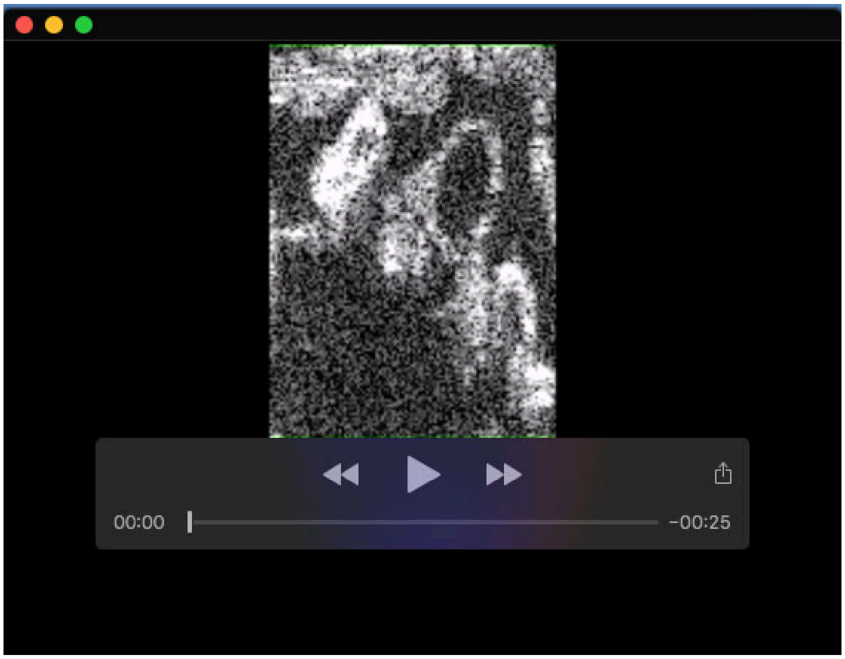

Movie 6. M-mode video of 9 day old Z^{24}; gZ^{HCM2}/+ beating adult heart. Video is captured around A1 segment.

Disease Models & Mechanisms • Supplementary information
